# Genomic Variants Associated with Haematological Parameters and T Lymphocyte Subpopulations in a Large White and Min Pig Intercross Population

**DOI:** 10.3390/ani14213140

**Published:** 2024-11-01

**Authors:** Naiqi Niu, Runze Zhao, Ming Tian, Wencheng Zong, Xinhua Hou, Xin Liu, Ligang Wang, Lixian Wang, Longchao Zhang

**Affiliations:** 1 State Key Laboratory of Animal Biotech Breeding, Institute of Animal Sciences, Chinese Academy of Agricultural Sciences (CAAS), Beijing 100193, China; caasnnq@126.com (N.N.); zhaorunze08@163.com (R.Z.); zongzone@outlook.com (W.Z.); 7hxh73@163.com (X.H.); firstliuxin@163.com (X.L.); ligwang@126.com (L.W.); iaswlx@263.net (L.W.); 2 Institute of Animal Husbandry, Heilongjiang Academy of Agricultural Sciences, Harbin 150086, China; tianming@haas.cn

**Keywords:** genome-wide association study, CD4+/CD8+, CD4-CD8+CD3+, platelet count, SNP

## Abstract

The breeding of disease-resistant pigs has consistently been a topic of significant interest and concern within the pig farming industry. The study of pig blood indicators has the potential to confer economic benefits upon the pig farming industry, whilst simultaneously providing valuable insights that can inform the study of human diseases. In this study, an F2 resource population of 489 individuals was generated through the intercrossing of Large White boars and Min pig sows. A total of 17 haematological parameters and T lymphocyte subpopulations were measured, including white blood cell count (WBC), lymphocyte count (LYM), lymphocyte count percentage (LYM%), monocyte count (MID), monocyte count percentage (MID%), neutrophilic granulocyte count (GRN), percentage of neutrophils (GRN%), mean platelet volume (MPV), platelet distribution width (PDW), platelet count (PLT), CD4+/CD8+, CD4+CD8+CD3+, CD4+CD8−CD3+, CD4−CD8+CD3+, CD4−CD8−CD3+, and CD3+. The Illumina PorcineSNP60 Genotyping BeadChip was obtained for all of the F2 animals. Subsequently, a genome-wide association study (GWAS) was conducted using the TASSEL 5.0 software to identify associated variants and candidate genes for the 17 traits. Significant association signals were identified for PCT and PLT on SSC7, with 1 and 11 significant SNP loci, respectively. A single nucleotide polymorphism (SNP) on SSC12 was identified as a significant predictor of the white blood cell (WBC) trait. Significant association signals were detected for the T lymphocyte subpopulations, namely CD4+/CD8+, CD4+CD8+CD3+, CD4+CD8−CD3+, and CD4−CD8+CD3+, with the majority of these signals observed on SSC7. The genes *CLIC5*, *TRIM15*, and *SLC17A4* were identified as potential candidates for influencing CD4+/CD8+ and CD4−CD8+CD3+. A missense variant, c.2707 G>A, in the SLC17A4 gene has been demonstrated to be significantly associated with the CD4+/CD8+ and CD4-CD8+CD3+ traits. Three missense variants (c.425 A>C, c.500 C>T, and c.733 A>G) have been identified in the TRIM15 gene as being linked to the CD4+/CD8+ trait. Nevertheless, only c.425 A>C has been demonstrated to be significantly associated with CD4-CD8+CD3+. In the *CLIC5* gene, one missense variant (c.957 T>C) has been identified as being associated with the CD4+/CD8+ and CD4-CD8+CD3+ traits. Additionally, significant association signals were observed for CD4+CD8+CD3+ and CD4+CD8−CD3+ on SSC2 and 5, respectively. Subsequently, a gene ontology (GO) enrichment analysis was conducted on all genes within the quantitative trait loci (QTL) intervals of platelet count, CD4+/CD8+, and CD4−CD8+CD3+. The MHC class II protein complex binding pathway was identified as the most significant pathway among the three immune traits. These results provide guidance for further research in the field of breeding disease-resistant pigs.

## Simple Summary

A genome-wide association study (GWAS) was conducted on 489 F2 pigs from a cross between Large White boars and Min pig sows, focusing on haematological parameters and T lymphocyte subpopulations (17 traits). Using the Illumina PorcineSNP60 Genotyping BeadChip, significant SNP loci were identified, particularly on *Sus scrofa* chromosome 7 (SSC7) for platelet count (PLT) and plateletocrit (PCT), with glutamate metabotropic receptor 4 (*GRM4*) as a candidate gene for PLT. White blood cell count (WBC) showed a significant association with SSC12. For T lymphocyte traits, significant signals were found on SSC7, SSC2, and SSC5, with candidate genes including, chloride intracellular channel 5 (*CLIC5*), tripartite motif containing 15 (*TRIM15*), and solute carrier family 17 member 4 (*SLC17A4*). Gene ontology (GO) enrichment analysis highlighted the MHC class II protein complex binding pathway as the most significant for immune traits. These findings offer valuable insights for breeding disease-resistant pigs and understanding immune-related traits in both pigs and potentially humans.

## 1. Introduction

Disease in the pig industry has always been a key factor limiting breeding efficiency. These traits are complex quantitative traits and mostly have moderate to low heritability. These factors affect the study of immune-related traits in pigs. However, recent advances in studies on disease resistance in swine have predominantly focused on classical swine fever virus (CSFV) [1,2], porcine reproductive and respiratory syndrome virus (PRRSV) [3,4], foot and mouth disease virus (FMDV) [5], transmissible gastroenteritis coronavirus (TGEV) [4], pseudorabies virus (PRV) [6], and Porcine Stress Syndrome (PSS) [7]. The diseases caused by these various pathogens lead to a range of immunological phenotypes in individuals. This study primarily aims to assess and investigate haematological parameters and T lymphocyte subpopulations within pig populations.

Certain immune traits are regarded as potential biomarkers for assessing pig health. The observation of medium to high heritabilities (h^2^ 0.4–0.8) in some of these traits indicates the presence of genetic variability within these phenotypes [8,9,10]. The Min pig is an excellent Chinese local breed with high immunity and strong disease resistance [11]. Our research mainly focuses on the F2 experimental design produced by intercrossing Large White boars and Min pig sows to investigate 17 traits, including complete blood count and T lymphocyte subsets. Complete blood count primarily assesses the cellular components of blood, including red blood cells (commonly known as erythrocytes), white blood cells (commonly known as leukocytes), and platelets. By observing changes in quantity and morphology, we can determine diseases. T lymphocytes play a crucial role in mediating cellular immune responses and are involved in humoral immune responses induced by T-cell-dependent (TD) Ag. Based on the surface markers and functional characteristics of T cells, they can be divided into different subsets. Each T cell subset has its own functional characteristics and works in synergy to accomplish immune response reactions and regulatory functions. In our study, we conducted population-based assessments of these traits and identified key quantitative trait loci (QTLs) and candidate genes through whole-genome association analysis as well as gene functional annotation methods. These findings can provide guidance for future disease-resistant breeding efforts.

The primary objective of this study was to identify the genetic factors influencing immune-related traits in pigs. By analysing the extensive data collected from the F2 generation, researchers aimed to uncover the genetic mechanisms underlying these traits. This research provided valuable insights into the genetic improvement of pigs, offering a solid foundation for future breeding strategies and contributing to the advancement of animal genetics.

## 2. Materials and Methods

### 2.1. Ethics Statement

All experiments and procedures were carried out following the regulations from the Animal Care and Ethics Committee for Animal Experiments, Institute of Animal Science, Chinese Academy of Agricultural Sciences (Beijing, China).

### 2.2. Population and Phenotypic Data

The three-generation resource population was developed to study genetic traits by intercrossing Large White boars and Min pig sows. Sixteen Min pig sows were initially mated with four Large White boars, resulting in the F1 generation, with each boar siring 130, 218, 15, and 126 F2 offspring, respectively. The F1 generation was carefully managed to avoid half-sib and full-sib mating (a boar mates with more than four sows), ensuring genetic diversity within the population. From the F1 generation, nine boars and 46 sows were selected to produce the F2 offspring. Through a meticulously planned breeding programme, these F1 animals were mated, leading to the production of 489 F2 animals, distributed across 65 litters.

All animals involved in this study were housed and raised under controlled conditions at the experimental farm of the Institute of Animal Science, Chinese Academy of Agricultural Sciences. The average space allocation per pig is 1.2 square metres. The feed used is exclusively sourced from the finisher feed products of the DBN Group (Beijing, China). During the rearing process, the health status of the pigs is monitored daily, and measures are taken to ensure their health both before and after slaughter. This consistent environment was maintained to ensure the reliability and comparability of the data collected. At 240 ± 7 days of age, the F2 animals were slaughtered in order to achieve a market weight of 109.36 ± 16.33 kg.

### 2.3. Genotyping and Quality Control

Genomic DNA was isolated from ear tissue using standard methods [12] and genotyped with the Illumina PorcineSNP60 Genotyping BeadChip, containing 42,749 SNPs across 18 autosomes (Table 1). The data were quality controlled through PLINK v1.90 [13]. Quality criteria included minor allele frequency (>5%), sample call rate (>90%), and SNP call rate (>90%).

### 2.4. Genome-Wide Association Study

TASSEL 5.0 [13,14,15] was used to conduct a genome-wide association study (GWAS) based on a mixed linear model (Q + K). TASSEL software was used to compute the kinship matrix and the principal component analysis (PCA). In order to account for the impacts of the population structure, PCA was used to build the “Q” matrix on the genotyping data set. The kinship matrix (K) was then calculated to take the place of pedigrees. Sex, slaughter batch and the first three PCAs were fixed effects, whereas the polygenic genetic effect was a random effect. To calculate the additive and dominating effects, we utilised the TASSEL programme. In this investigation, a threshold of 1.17 × 10^−6^ (0.05/42,749), where 42,749 is the total number of relevant SNPs in the data set, was applied.

### 2.5. Assignment of Genes to Gene Ontology (GO) Pathways and Pathway Analyses

In this study, we performed Gene Ontology (GO) enrichment analysis on all genes within the significant region of SSC7 for three traits, including PLT, CD4+/CD8+, and CD4−CD8+CD3+. First, enrichment analysis was conducted using the R package clusterProfiler [16], and subsequently, bubble plots of the enrichment results were generated using the R package ggplot2.

### 2.6. Primer Design and Synthesis

A total of 22 primer pairs were designed for the *SLC17A4* (XM_021098408.1), *TRIM15* (NM_001123208.1), and *CLIC5* (NM_001198923.2) genes using the Primer Premier 5.0 software. The design process is illustrated in Supplementary Table S1. The primers were synthesised by Liuhe Huada Gene Technology Co. (Beijing, China).

### 2.7. Statistical Analysis

Following the sequencing of the PCR amplification products, genotyping was performed utilising the SeqMan 7.1module in DNAStar 7.1. Genotypic and allelic frequency statistics were calculated using Microsoft Excel 2016. The association between phenotypes and genotypes was analysed using SAS 9.2, and Duncan’s multiple range test was employed to assess the significance of differences between genotypes (*p* < 0.05). Data were presented as mean ± standard error, and a significance level of *p* < 0.05 was used to determine statistical significance.

## 3. Results

### 3.1. Phenotype Description

We collected data on 17 traits (WBC, LYM, LYM%, MID, MID%, GRN, GRN%, MPV, PDW, PCT, PLT, CD4+/CD8+, CD4+CD8+CD3+, CD4+CD8−CD3+, CD4−CD8+CD3+, CD4−CD8−CD3+ and CD3+), including haematological parameters and T lymphocyte subpopulations, from a resource population of 489 F2 animals. For each trait, we calculated key descriptive statistics: mean, standard deviation, maximum, and minimum values. These metrics provide a comprehensive overview of the phenotypic variation within the population. The detailed phenotypic values and their corresponding statistics are summarised and presented in Table 2, offering valuable insights into the genetic and physiological diversity observed in the study.

### 3.2. Genome-Wide Association Study of Haematological Parameters Traits

**Figure 1:**
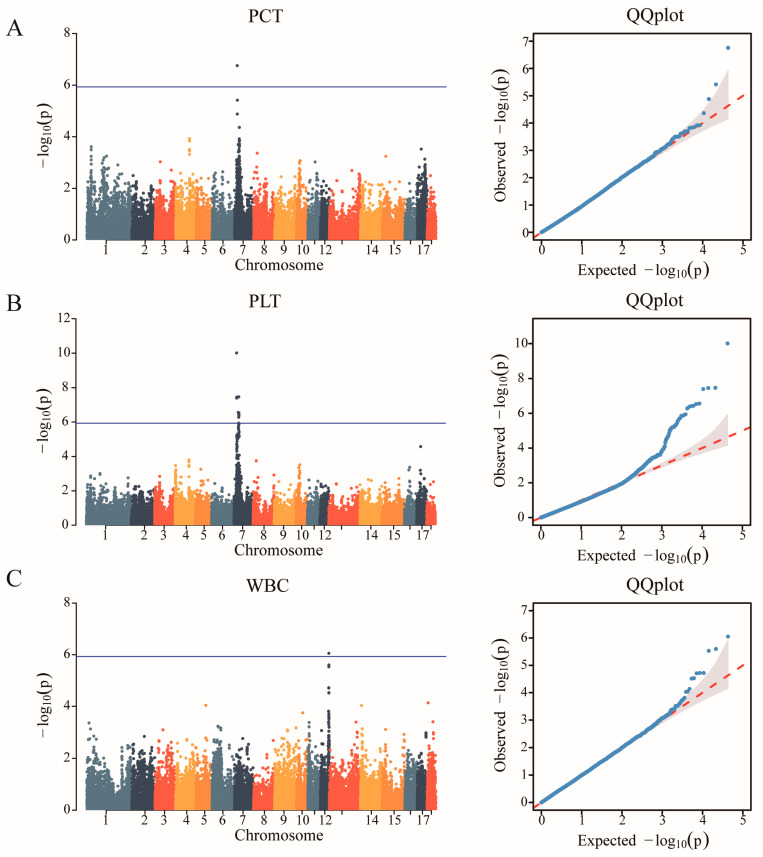
Identification of GWAS of the haematological parameters. (**A**) Manhattan plot displaying the GWAS results of plateletocrit (PCT). The blue horizontal line indicated the Bonferroni significance threshold (1.17 × 10^−6^). (**B**) Manhattan plot displaying the GWAS results of the platelet count (PLT). (**C**) Manhattan plot displaying the GWAS results of white blood cell count (WBC).

**Figure 2:**
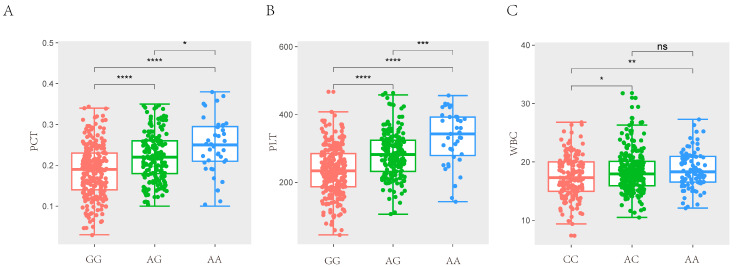
Comparison of three genotypes of significant SNP for haematological traits. (**A**) The difference analysis of MARC0014928 for PCT in SSC7. (**B**) The difference analysis of MARC0014928 for PLT in SSC7. (**C**) The difference analysis of ASGA0098229 for WBC in SSC12. (* *p* < 0.05, ** *p* < 0.01, *** *p* < 0.001, **** *p* < 0.0001, (*p* > 0.05, ns)).

After quality control, a total of 42,749 SNPs distributed across the 18 autosomes were selected. The final data set used for the analysis of the 11 haematological parameters traits (WBC, LYM, LYM%, MID, MID%, GRN, GRN%, MPV, PDW, PCT, and PLT) included 42,749 SNPs and 489 individuals from the F2 resource population. The Manhattan and Q-Q plots are shown in Figure 1, Supplementary Figures S1 and S2, respectively. A total of 12 SNPs that were genome-level significant with haematological characteristics were found in this investigation. As shown in Table 3, 1, 11, and 1 significant SNP loci were identified for PCT, PLT, and WBC, respectively. A comparison of the three genotypes at the most significant SNP loci associated with PCT, PLT, and WBC is presented in Figure 2.

### 3.3. Genome-Wide Association Study of T Lymphocyte Subpopulation Traits

**Figure 3:**
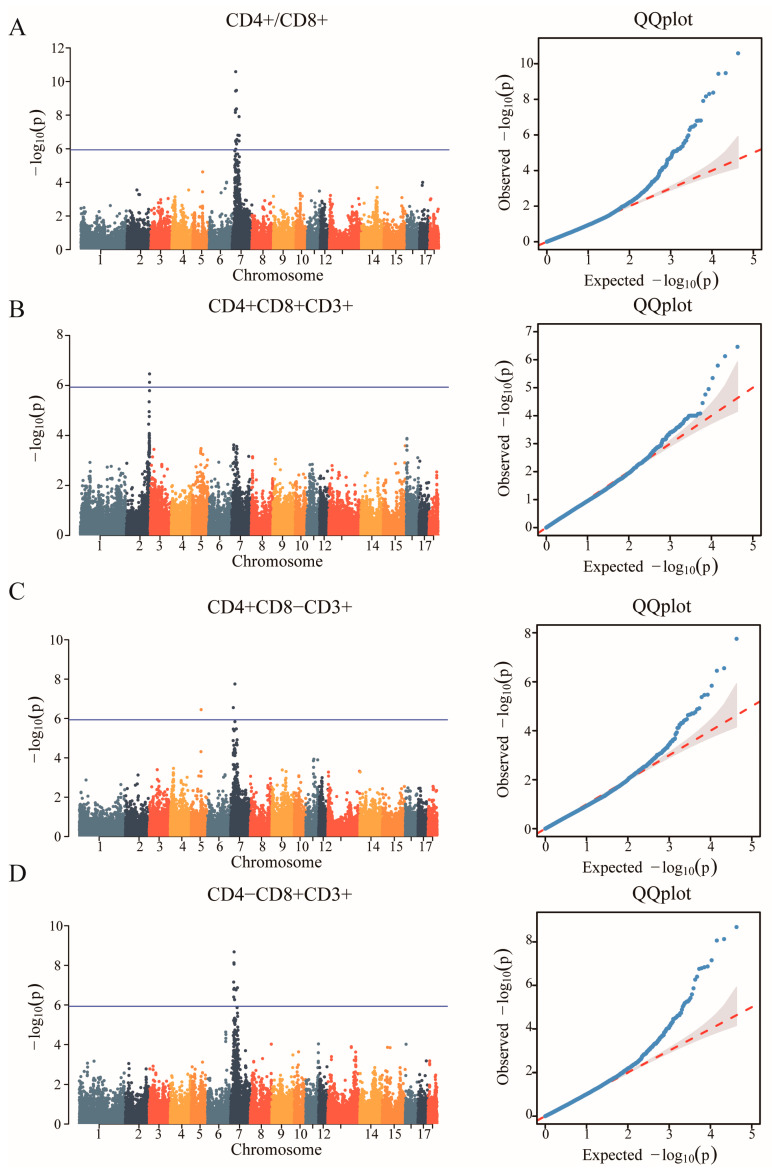
Identification of GWAS of the T lymphocyte subpopulation. (**A**) Manhattan plot displaying the GWAS results of the CD4+/CD8+. The blue horizontal line indicated the Bonferroni significance threshold (1.17 × 10^−6^). (**B**) Manhattan plot displaying the GWAS results of the CD4+CD8+CD3+. (**C**) Manhattan plot displaying the GWAS results of the CD4+CD8−CD3+. (**D**) Manhattan plot displaying the GWAS results of the CD4−CD8+CD3+.

**Figure 4:**
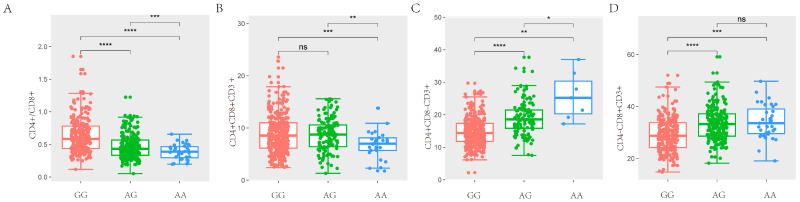
Comparison of three genotypes of significant SNP in T lymphocyte subpopulation traits. (**A**) The difference analysis of ASGA0031860 for CD4+/CD8+ in SSC7. (**B**) The difference analysis of ALGA0017071 for CD4+CD8+CD3+ in SSC2. (**C**) The difference analysis of ASGA0032099 for CD4+CD8−CD3+ in SSC7. (**D**) The difference analysis of ASGA0031860 for CD4−CD8+CD3+ in SSC7. (* *p* < 0.05, ** *p* < 0.01, *** *p* < 0.001, **** *p* < 0.0001, (*p* > 0.05, ns)).

In the study, we obtained a total of 42,749 SNPs distributed across the 18 autosomes. For the six T lymphocyte subpopulation traits (CD4+/CD8+, CD4+CD8+CD3+, CD4+CD8−CD3+, CD4−CD8+CD3+, CD4−CD8−CD3+ and CD3+), the Manhattan and Q-Q plots are shown in Figure 3, and Supplementary Figure S3, respectively. A total of 23 genome-wide significant SNPs that were genome-level significant with T lymphocyte subpopulation characteristics were found in this investigation. With regard to two characteristics, including CD3, and CD4-CD8-CD3+, there were no significant SNPS. On the other hand, 15, 2, 3, and 9 significant SNP locations, respectively, were found for the remaining three characteristics CD4+/CD8+, CD4+CD8+CD3+, CD4+CD8−CD3+ and CD4−CD8+CD3+, as shown in Table 4. A comparison of the three genotypes at the most significant SNP loci for CD4+/CD8+, CD4+CD8+CD3+, CD4+CD8−CD3+, and CD4−CD8+CD3+ was conducted in Figure 4.

### 3.4. GO Analysis

**Figure 5:**
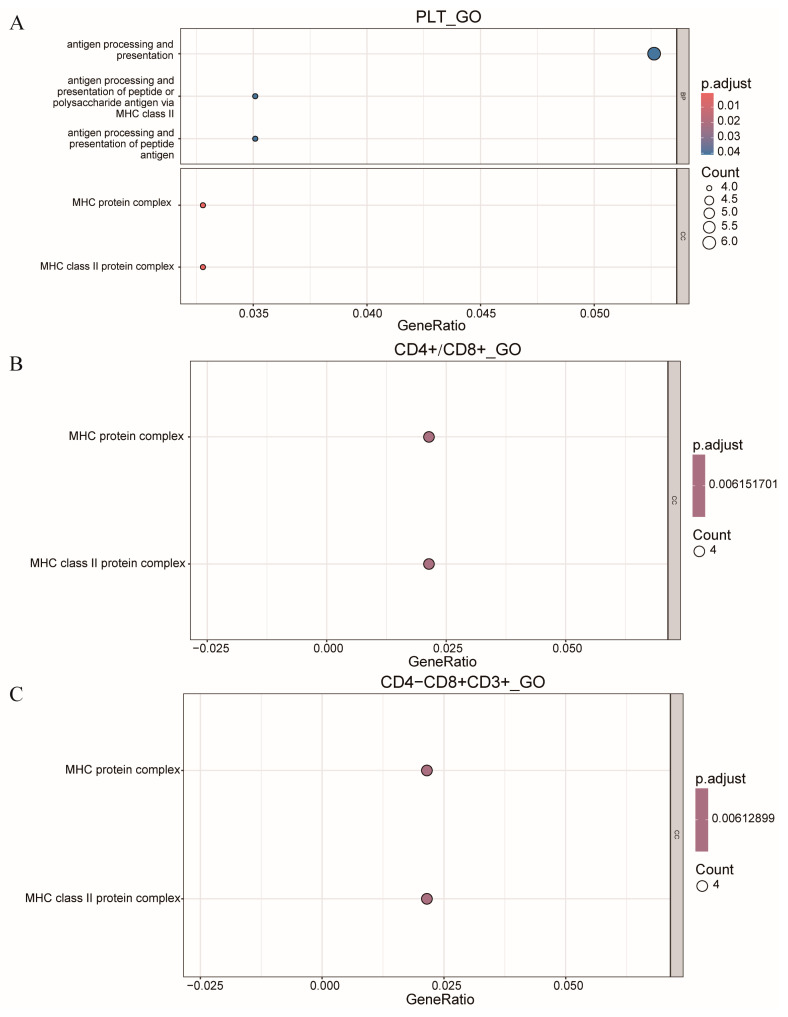
Bubble chart of GO function enrichment analysis of genes in SSC7. (**A**) PLT of GO function enrichment analysis. (**B**) CD4+/CD8+ of GO function enrichment analysis. (**C**) CD4−CD8+CD3+ of GO function enrichment analysis.

In this study, we performed enrichment analysis on all genes located within the QTLs on SSC7, specifically for the traits PLT, CD4+/CD8+, and CD4−CD8+CD3+. The results, presented in Figure 5, revealed the identification of two common peaks, both within the MHC protein complex and the MHC class II protein complex. These findings suggest that these regions may play a significant role in the regulation of these immune-related traits, providing important insights into the genetic architecture underlying these characteristics.

### 3.5. Statistical Analysis of Data

The CDS regions of three genes, *SLC17A4*, *TRIM15*, and *CLIC5*, were amplified by polymerase chain reaction (PCR), resulting in the identification of six missense variants (two in *SLC17A4*, three in *TRIM15*, and one in *CLIC5*). The statistical analysis of these variants in relation to CD4+/CD8+ and CD4−CD8+CD3+ is presented in Table 5. A missense variant in the *SLC17A4* gene (c.2707 G>A) is considered to be significantly associated with the CD4+/CD8+ and CD4−CD8+CD3+ traits. In the *TRIM15* gene, three missense variants (c.425 A>C, c.500 C>T, and c.733 A>G) were found to be significantly associated with the CD4+/CD8+ trait. Nevertheless, only the c.425 A>C variant demonstrates a statistically significant correlation with the CD4−CD8+CD3+ trait. One missense variant in the *CLIC5* gene (c.957 T>C) is significantly associated with both the CD4+/CD8+ and CD4−CD8+CD3+ traits.

## 4. Discussion

Previous studies have consistently identified significant SNPs associated with WBC counts on SSC3 [17] and SSC5 [18], highlighting these regions as critical for WBC regulation. However, the present study has identified a significant SNP, ASGA0098229, on chromosome SSC12 that influences the WBC trait. This novel finding suggests that SSC12 may have a previously underappreciated role in WBC regulation. Conversely, although significant SNPs for LYM counts have been previously observed on SSC5 [18], our study did not detect any significant SNPs associated with the LYM trait. This discrepancy could be due to differences in study designs, populations, or underlying genetic factors influencing LYM counts, indicating the need for further research to elucidate the genetic determinants of LYM variability.

The study of immune traits is currently a hot topic and a challenge in the field of disease-resistant breeding in pigs, due to the numerous factors that affect it (sampling time points, measurement methods, accuracy of measurements, etc.), as well as the relatively high cost of measurements compared to other traits. Furthermore, the QTL mapping results for immune-related traits may vary at different time points [19,20]. In this study, we conducted a genome-wide association analysis for 17 immune traits, including blood cell parameters and T lymphocyte subpopulations, in adult pigs. Significant association signals were mainly found on chromosomes 2, 5, 7, and 12. Additionally, significant association signals were found on chromosome 7 for five traits, including PCT, PLT, CD4+/CD8+, CD4+CD8−CD3+, and CD4−CD8+CD3+.

In the case of the traits CD4+/CD8+ and CD4−CD8+CD3+, a single locus exerts influence over multiple traits simultaneously. The ASGA0031860 locus is the most significant single-nucleotide polymorphism (SNP) identified for both traits, although this locus is not an intragenic variant. Furthermore, a number of additional shared SNP loci were also identified. MARC0015432 was localised on the *CLIC5*, which has been demonstrated to serve as an independent prognostic factor for lung cancer and to be closely associated with the infiltration levels of various immune cells and immune markers [21]. Subsequently, the coding sequence region of the *CLIC5* gene was screened for variations, resulting in the identification of six missense variants. The missense variant (c.957 T>C) is worthy of particular attention, involving a change from threonine to alanine, with the Ensembl database indicating a 100% probability of a functional change resulting from this substitution. MARC0055565 was identified as being located on the *TRIM15* gene, which is a member of the E3 ubiquitin ligase gene family. This gene family is tightly clustered from HLA-E to HLA-A, mainly within the MYH class I gene cluster. The *TRIM15* gene is associated with immune response. This is evidenced by reference [22]. A missense variant was identified in the *TRIM15* gene (c.425 A>C), which demonstrated a significant correlation with the CD4+/CD8+ and CD4−CD8+CD3+ traits. The amino acid substitution involves an aspartic acid to alanine (D/A) change, with the Ensembl database indicating that there is a 28% probability of a functional change resulting from this variant. The locus ALGA0039343 was identified within the *SLC17A4* gene, also known as *NPT5*, which is a member of the SLC17 phosphate transporter protein family and is an organic anion transporter in the intestine [23]. The results of studies conducted in different populations have been inconsistent. For instance, the polymorphism of *SLC17A4* has been associated with gout in Chinese patients [24] but not in New Zealanders [25]. A missense variant was identified in the *SLC17A4* gene (c.2707 G>A), which demonstrated a significant correlation with the CD4+/CD8+ and CD4−CD8+CD3+ traits. The amino acid substitution involves a valine to isoleucine (V/I) change, with the Ensembl database indicating that there is a 93% probability of a functional change resulting from this variant. Nevertheless, there is currently no direct evidence to substantiate the hypothesis that the genes in question are associated with the traits CD4+/CD8+ and CD4−CD8+CD3+, as investigated in this research project. Accordingly, further experimental validation is required to confirm this relationship.

For the trait PLT, we are interested in the *GRM4* near the MARC0058766 locus. It is a type III metabotropic glutamate receptor that is highly expressed in the central nervous system. Multiple studies have shown its involvement in various physiological and pathophysiological processes such as learning, memory, and cognitive impairment. However, there are also reports of *GRM4* expression in multiple immune cells [26,27,28]. In mouse experiments, it was found that knockout of *GRM4* made mice highly susceptible to experimental autoimmune encephalomyelitis (EAE), a mouse model of multiple sclerosis. In cell experiments, it was observed that knockout of *GRM4* in dendritic cells promoted polarisation of inactive CD4+ T cells into T-helper 17 cells (TH17 cells) producing interleukin-17 (IL-17), leading to the progression of EAE [27]. However, there is no research indicating the association between *GRM4* and PLT, which requires further validation.

Based on the above analysis results, we conducted enrichment analysis on the genes in the QTLs on SSC7 for the three traits, PLT, CD4+/CD8+, and CD4−CD8+CD3+. It was found that all three traits were enriched in the cellular component of the MYH protein complex and MHC class II protein complex in the GO enrichment analysis. The MHC class II protein complex is an important protein in the immune system, playing a crucial role in antigen presentation and immune response processes. The T cell arm of the adaptive immune response has evolved to specifically recognise the products resulting from partial intracellular proteolysis. CD4+ T cells specifically recognise peptides that are bound to major histocompatibility complex class II (MHC-II) molecules [29].

## 5. Conclusions

In this study, we conducted GWAS and GO analysis on 17 haematological parameters and T lymphocyte subpopulations. The GWAS results demonstrated that PCT, PLT, CD4+/CD8+, CD4+CD8−CD3+, and CD4−CD8+CD3+ exhibited notable association signals on SSC7, whereas WBC, CD4+CD8+CD3+, and CD4+CD8−CD3+ displayed significant association signals on SSC12, SSC2, and SSC5, respectively. The *GRM4* gene was identified as a potential candidate gene influencing PLT. The *CLIC5*, *TRIM15*, and *SLC17A4* genes were identified as potential candidate genes influencing CD4+/CD8+ and CD4−CD8+CD3+. A missense variant c.2707 G>A in the *SLC17A4* gene has been identified as significantly associated with the CD4+/CD8+ and CD4−CD8+CD3+ traits. In the *TRIM15* gene, three missense variants (c.425 A>C, c.500 C>T, and c.733 A>G) are linked to the CD4+/CD8+ trait, while only the variant c.425 A>C is also significantly associated with CD4−CD8+CD3+. The *CLIC5* gene has one missense variant (c.957 T>C) significantly associated with the CD4+/CD8+ and CD4−CD8+CD3+. Subsequently, GO analysis of all genes associated with platelet count (PLT), CD4+/CD8+ T-cell ratio, and CD4−CD8+CD3+ quantitative trait loci (QTLs) revealed an enrichment of the MHC class II protein complex binding pathway. These findings provide valuable insights for future research in the domain of breeding disease-resistant pigs.

## Figures and Tables

**Table 1:** Distribution of SNPs after quality control and average distances on each chromosome.

Chr ^1^	SNPs	Average Distance (kb)
1	5255	52.20
2	2739	55.47
3	2253	58.97
4	2908	45.02
5	1960	53.33
6	2614	65.36
7	2777	43.88
8	2284	60.84
9	2725	51.20
10	1473	47.09
11	1569	50.46
12	1322	46.60
13	3371	61.80
14	3085	45.95
15	2401	58.48
16	1547	51.68
17	1366	46.48
18	1100	50.89
Total	42,749	

^1^ Chr, Chromosome.

**Table 2:** Statistics of phenotypic values of the number of 11 Haematological parameters and 6 T lymphocyte subpopulation for 489 population.

Traits	Mean	Standard Deviation	Maximum	Minimum
WBC	18.13	3.49	31.80	7.40
PLT	261.01	76.63	467	45.00
PCT	0.20	0.06	0.38	0.03
MPV	8.05	1.16	11.70	5.10
PDW	14.49	0.78	17.20	12.00
LYM	11.79	2.58	20.00	4.40
MID	2.24	0.86	5.20	0.80
GRN	4.10	2.48	15.50	0.20
LYM%	65.33	9.70	84.70	36.30
MID%	12.44	4.49	27.60	4.80
GRN%	22.22	11.51	52.80	1.20
CD4−CD8−CD3+	23.70	7.91	46.70	4.60
CD4+CD8−CD3+	15.97	5.04	37.70	2.20
CD4−CD8+CD3+	31.43	6.80	59.10	14.80
CD4+CD8+CD3+	8.75	3.54	23.60	1.30
CD3	79.86	5.61	94.30	57.30
CD4+/CD8+	0.54	0.24	1.85	0.05

Note: white blood cell count (WBC, ×10^9^/L), lymphocyte count (LYM, ×10^9^/L), lymphocyte count percentage (LYM%, %), monocyte count (MID, ×10^9^/L), monocyte count percentage (MID%, %), neutrophilic granulocyte count (GRN, ×10^9^/L), neutrophilic granulocyte percentage (GRN%, %), mean platelet volume (MPV, fL), platelet distribution width (PDW, ×10^9^/L), plateletocrit (PCT, mL/L), platelet count (PLT, ×10^9^/L), CD4+/CD8+, CD4+CD8+CD3+, CD4+CD8−CD3+, CD4−CD8+CD3+, CD4−CD8−CD3+ and CD3+.

**Table 3:** Genome-wise significant SNP for haematological traits.

Traits	SNP	Alleles	Chr ^1^	Position ^2^	Nearest Genes	Distance ^3^ (bp)	*p* Value
PCT	MARC0014928	C/T	7	18186149	*ENSSSCG00000054723*	34,111	1.76 × 10^−7^
PLT	MARC0014928	C/T	7	18186149	*ENSSSCG00000054723*	34,111	9.75 × 10^−11^
	ALGA0040148	T/C	7	29487703	*COL21A1*	within	3.40 × 10^−8^
	ALGA0039151	T/C	7	19039982	*DCDC2*	8106	3.53 × 10^−8^
	ASGA0031602	T/C	7	18666443	*ENSSSCG00000057284*	17,358	4.02 × 10^−8^
	MARC0087333	A/G	7	25830498	*HCRTR2*	within	2.75 × 10^−7^
	MARC0058766	C/T	7	30144081	*GRM4*	35,224	2.94 × 10^−7^
	H3GA0020692	A/G	7	29338532	*DST*	38,380	3.71 × 10^−7^
	H3GA0020765	T/G	7	30095798	*ENSSSCG00000047035*	26,926	3.85 × 10^−7^
	MARC0033464	C/T	7	30572315	*ILRUN*	within	4.27 × 10^−7^
	ASGA0032322	A/G	7	28798509	*BEND6*	within	5.26 × 10^−7^
	ALGA0040120	G/A	7	29289201	*DST*	within	1.15 × 10^−6^
WBC	ASGA0098229	A/C	12	57522622	*ENSSSCG00000059067*	69,298	8.82 × 10^−7^

^1^ Chr, Chromosome; ^2^ Data from Sus scrofa Build 11.1; ^3^ SNP designated as in a gene or distance (bp) from a gene region.

**Table 4:** Genome-wise significant SNP for T lymphocyte subpopulation traits.

Traits	SNP	Alleles	Chr ^1^	Position ^2^	Nearest Genes	Distance ^3^ (bp)	*p* Value
CD4+/CD8+	ASGA0031860	G/A	7	22075114	*ZSCAN9*	7057	2.60 × 10^−11^
	ASGA0032099	C/T	7	24944510	*ENSSSCG00000001456*	8984	3.37 × 10^−10^
	ALGA0039770	A/G	7	25014857	*ENSSSCG00000063263*	11,156	4.21 × 10^−9^
	ASGA0031822	A/C	7	21349121	*ENSSSCG00000059172*	within	4.98 × 10^−9^
	ALGA0039343	C/T	7	20515855	*SLC17A4*	within	6.89 × 10^−9^
	MARC0015432	A/G	7	40842218	*CLIC5*	within	1.23 × 10^−8^
	INRA0024930	T/G	7	34123154	*BTBD9*	within	1.56 × 10^−7^
	H3GA0020975	T/C	7	34136279	*BTBD9*	11,821	1.56 × 10^−7^
	ALGA0041094	G/A	7	41580629	*ENSSSCG00000001724*	7120	1.62 × 10^−7^
	ASGA0032255	G/A	7	27765368	*KHDRBS2*	within	2.82 × 10^−7^
	H3GA0055194	A/G	7	21926288	*ENSSSCG00000054486*	within	3.37 × 10^−7^
	ALGA0041025	C/T	7	40830444	*CLIC5*	within	3.46 × 10^−7^
	MARC0055565	G/A	7	22727959	*TRIM15*	within	3.80 × 10^−7^
	DRGA0007396	C/T	7	25565917	*ENSSSCG00000055495*	16,872	5.21 × 10^−7^
	MARC0006637	A/C	7	22951243	*ENSSSCG00000041364*	within	1.06 × 10^−6^
CD4+CD8+CD3+	ALGA0017071	C/T	2	151238895	*SLC6A7*	within	3.46 × 10^−7^
	ASGA0012938	C/A	2	151287051	*CAMK2A*	within	7.50 × 10^−7^
CD4+CD8−CD3+	ASGA0032099	C/T	7	24944510	*ENSSSCG00000001456*	8984	1.76 × 10^−8^
	ASGA0031549	T/C	7	17257230	*ENSSSCG00000049691*	within	2.80 × 10^−7^
	MARC0028162	A/G	5	65032951	*NTF3*	19,568	3.55 × 10^−07^
CD4−CD8+CD3+	ASGA0031860	G/A	7	22075114	*ZSCAN9*	7057	2.09 × 10^−9^
	ASGA0031822	A/C	7	21349121	*ENSSSCG00000059172*	within	7.45 × 10^−9^
	ASGA0083507	A/G	7	19971763	*CMAH*	16,004	7.00 × 10^−8^
	MARC0015432	A/G	7	40842218	*CLIC5*	within	1.35 × 10^−7^
	MARC0055565	G/A	7	22727959	*TRIM15*	within	1.46 × 10^−7^
	ALGA0039343	C/T	7	20515855	*SLC17A4*	within	1.62 × 10^−7^
	ASGA0032668	A/G	7	32640406	*MTCH1*	4831	1.74 × 10^−7^
	ALGA0039300	A/G	7	20253597	*CARMIL1*	within	3.98 × 10^−7^
	ALGA0039634	T/G	7	23504078	*C6orf15*	11,559	5.43 × 10^−7^

^1^ Chr, chromosome. ^2^ Data from Sus scrofa Build 11.1. ^3^ SNP designated as in a gene or distance (bp) from a gene region.

**Table 5:** The association analysis of six SNPs of *SLC17A4*, *TRIM15*, and *CLIC5* gene with CD4+/CD8+ and CD4−CD8+CD3+.

SNP ^1^	Gene	Amino Acids	Number	Genotypes	CD4+/CD8+	CD4−CD8+CD3+
c.2696 C>T	*SLC17A4*	A/V	1	TT	0.40	34.20
			45	TC	0.44 ± 0.03	34.20 ± 1.21
			360	CC	0.57 ± 0.01	30.91 ± 0.34
c.2707 G>A	*SLC17A4*	V/I	15	AA	0.79 ± 0.10 ^a^	27.54 ± 1.42 ^b^
			144	AG	0.65 ± 0.02 ^b^	29.45 ± 0.55 ^ab^
			282	GG	0.49 ± 0.01 ^c^	32.24 ± 0.38 ^a^
c.425 A>C	*TRIM15*	D/A	12	CC	0.90 ± 0.13 ^a^	25.73 ± 1.30 ^b^
			70	CA	0.71 ± 0.03 ^b^	29.44 ± 0.77 ^ab^
			378	AA	0.50 ± 0.01 ^c^	32.04 ± 0.35 ^a^
c.500 C>T	*TRIM15*	A/V	5	TT	0.61 ± 0.11 ^ab^	34.22 ± 1.71
			56	TC	0.74 ± 0.04 ^a^	29.12 ± 0.85
			398	CC	0.52 ± 0.01 ^b^	31.71 ± 0.35
c.733 A>G	*TRIM15*	T/A	2	GG	1.05 ± 0.01 ^a^	23.65 ± 3.75
			59	GA	0.56 ± 0.03 ^b^	29.35 ± 0.93
			410	AA	0.54 ± 0.01 ^b^	31.79 ± 0.33
c.957 T>C	*CLIC5*	T/A	34	TT	0.67 ± 0.05 ^a^	29.26 ± 0.91 ^b^
			83	TC	0.61 ± 0.03 ^a^	30.93 ± 0.70 ^ab^
			321	CC	0.51 ± 0.01 ^b^	31.76 ± 0.39 ^b^

^1^ SNP: Location of the variant on the mRNA. Note: In the same SNP and column, values with different upper indices indicate a significant difference (*p* < 0.05); however, with same or no upper indices indicate no significant difference (*p* > 0.05).

## Data Availability

The data presented in this study are available on request from the corresponding author.

## Supplementary Materials

The following supporting information can be downloaded at: https://www.mdpi.com/article/10.3390/ani14213140/s1, Figure S1: Identification of GWAS of the haematological parameters; Figure S2: Identification of GWAS of the haematological parameters; Figure S3: Identification of GWAS of the T lymphocyte subpopulation; Table S1: Information of primer.
