# Supplementary material for: Genomic Variants Associated with Haematological Parameters and T Lymphocyte Subpopulations in a Large White and Min Pig Intercross Population
Source: Animals (Basel). 2024 Nov 1;14(21):3140. doi: 10.3390/ani14213140 (PMC11545393; doi:10.3390/ani14213140)
Supplement: Supplementary file 1 [file animals-14-03140-s001.zip › Supplementary information.pdf]

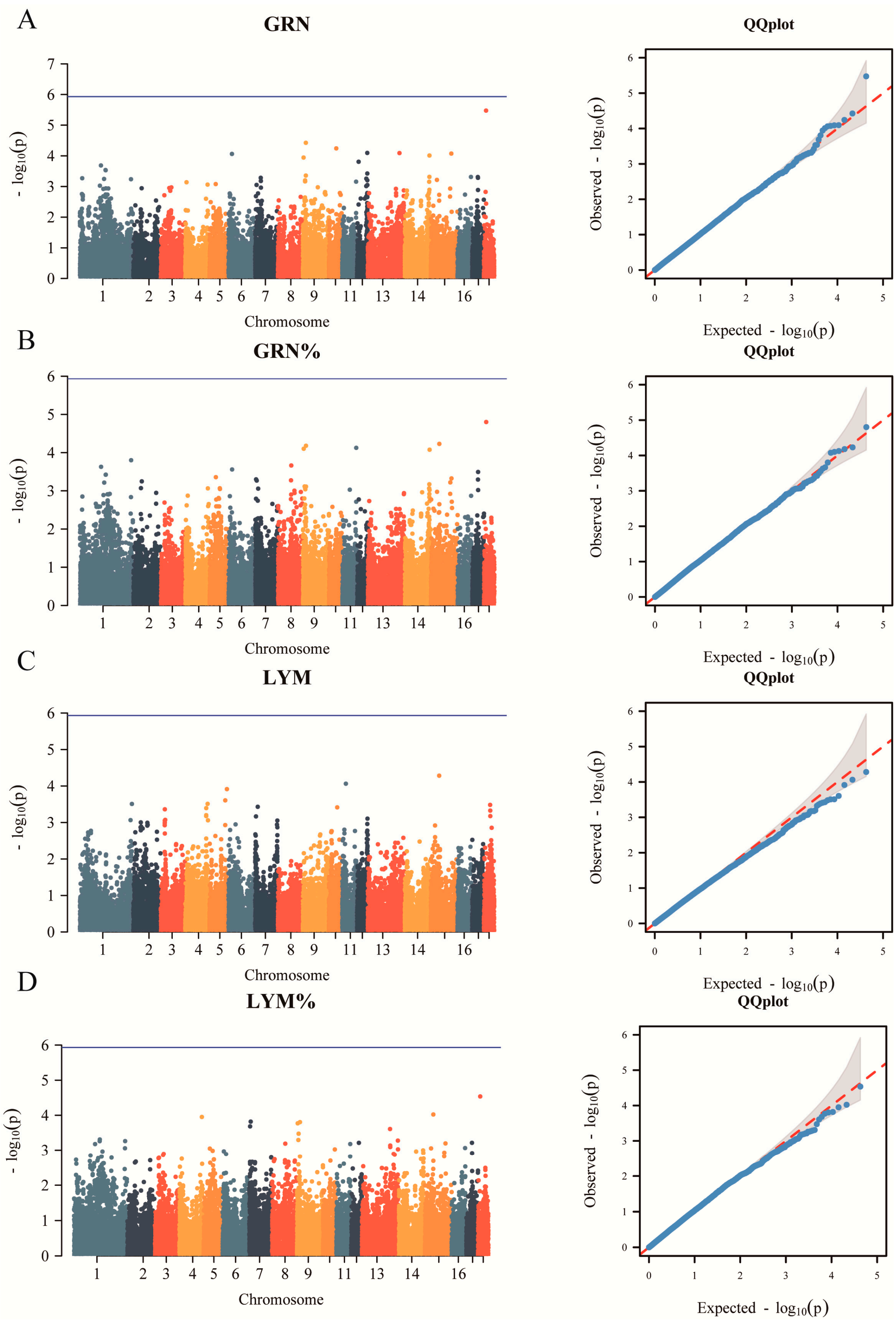

Figure S1 Identification of GWAS of the haematological parameters. (A) Manhattan plot displaying the GWAS results of neutrophilic granulocyte count (GRN). The red horizontal line indicated the Bonferroni significance threshold (1.17 × 10−6). (B) Manhattan plot displaying the GWAS results of the neutrophilic granulocyte percentage (GRN%). (C) Manhattan plot displaying the GWAS results of lymphocyte count (LYM). (D) Manhattan plot displaying the GWAS results of lymphocyte count percentage (LYM%).

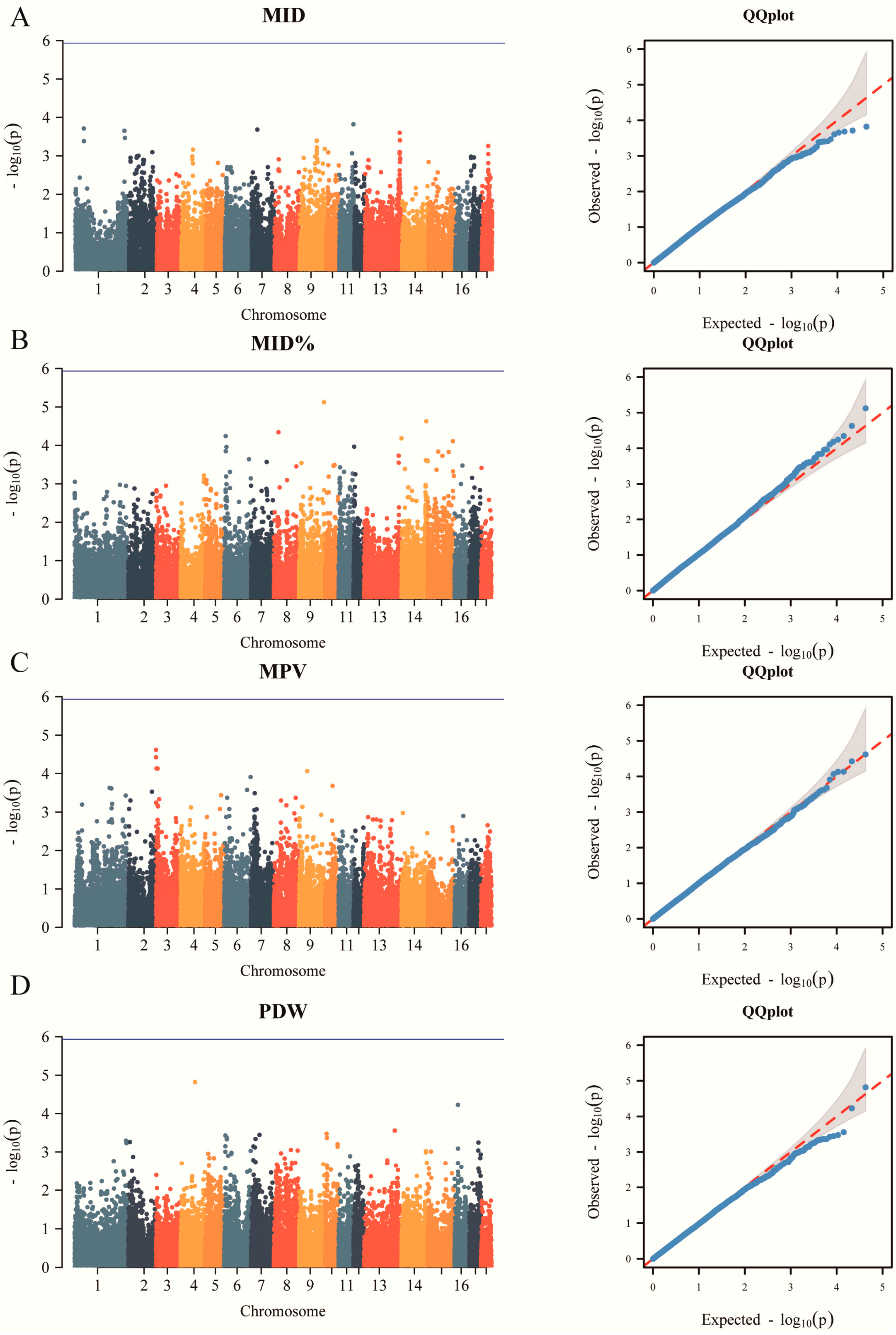

Figure S2 Identification of GWAS of the haematological parameters. (A) Manhattan plot displaying the GWAS results of monocyte count (MID). The red horizontal line indicated the Bonferroni significance threshold (1.17 × 10−6). (B) Manhattan plot displaying the GWAS results of the monocyte count percentage (MID%). (C) Manhattan plot displaying the GWAS results of mean platelet volume (MPV). (D) Manhattan plot displaying the GWAS results of platelet distribution width (PDW).

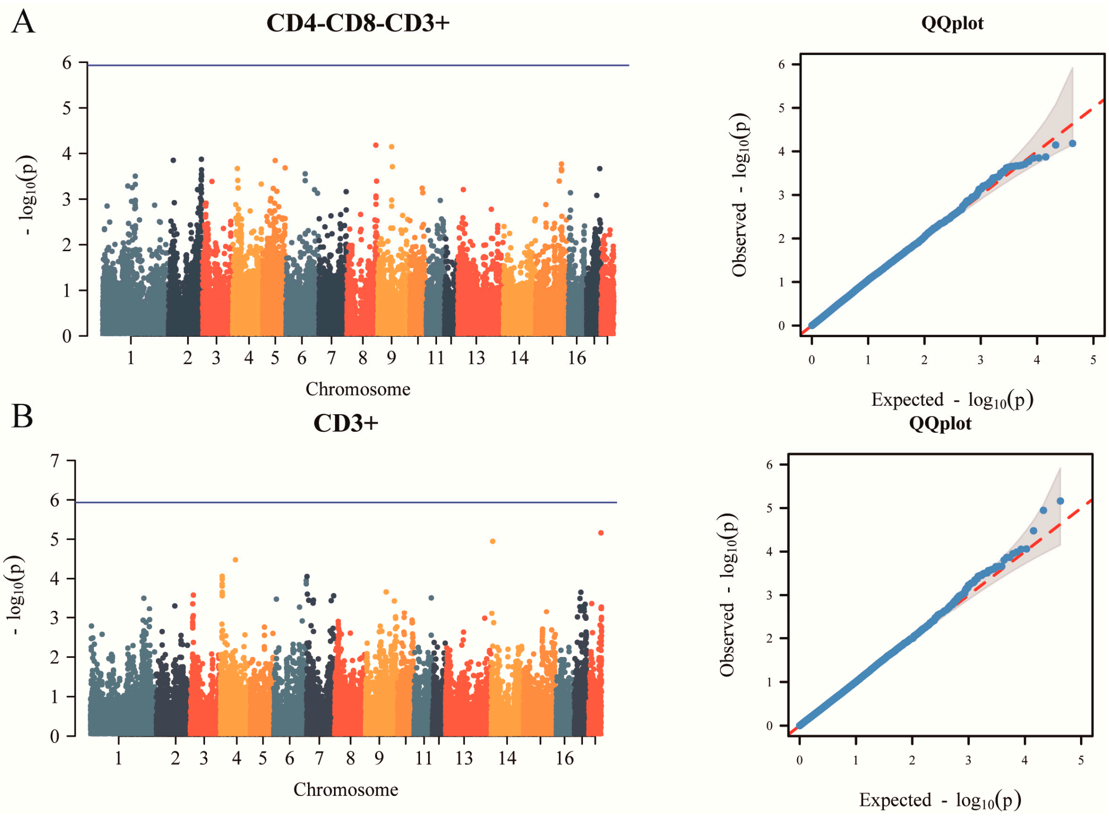

Figure S3 Identification of GWAS of the T lymphocyte subpopulation. (A) Manhattan plot displaying the GWAS results of the CD4-CD8-CD3+. The red horizontal line indicated the Bonferroni significance threshold (1.17 × 10−6). (B) Manhattan plot displaying the GWAS results of the CD3+.

Table S1 Information of primer.
